# Pesticides and Autism Spectrum Disorders: New Findings from the CHARGE Study

**DOI:** 10.1289/ehp.122-A280

**Published:** 2014-10-01

**Authors:** David C. Holzman

**Affiliations:** David C. Holzman writes on science, medicine, energy, economics, and cars from Lexington and Wellfleet, MA. His work has appeared in *Smithsonian*, *The Atlantic Monthly*, and the *Journal of the National Cancer Institute*.

A small but growing body of literature reports associations between pesticide exposures during pregnancy and characteristics of autism spectrum disorders (ASDs) or actual autism diagnosis.^1^^,^^2^^,^^3^ A study published this month in *EHP* adds to the weight of this evidence, reporting an increased risk of ASD diagnosis among children whose mothers lived during pregnancy near fields where pesticides were applied.^4^

The case–control study included 486 children diagnosed with an ASD, 168 diagnosed with delayed development, and 316 controls from the ongoing Childhood Autism Risks from Genes and Environment (CHARGE) study, which was launched in 2003. The researchers assessed timing and extent of pesticide applications within 1.75 km of each mother’s residence from 3 months before conception through the time of delivery. These data came from California’s Pesticide Use Report, which since 1990 has documented pesticide applications to farmland, golf courses, cemeteries, and other sites down to the square mile.^5^

**Figure:**
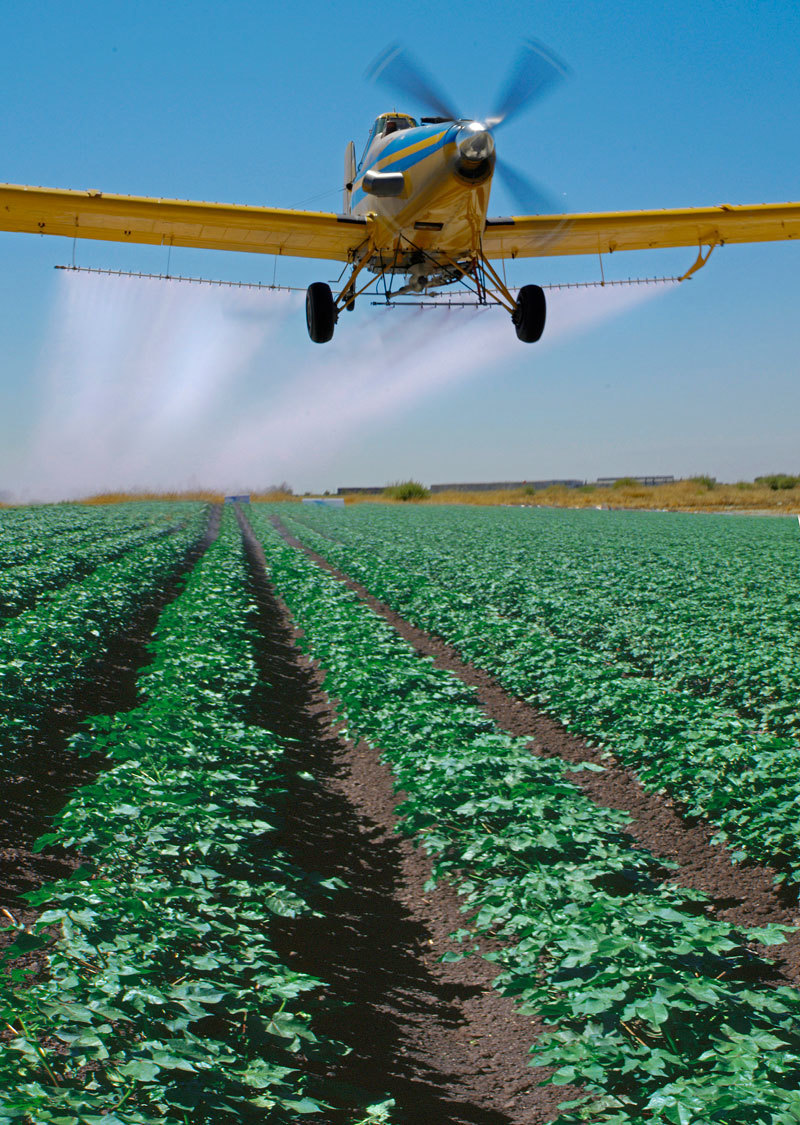
A new study suggests agricultural pesticides may be appropriate candidates for further study as autism risk factors. © Tony Hertz/AgStock Images/Corbis

The study examined the association between prenatal proximity to fields where organophosphate, pyrethroid, or carbamate pesticides were applied and later diagnosis of neurodevelopmental disorders including ASDs and developmental delays. The authors found the strongest associations between ASDs and application of nonspecified organophosphates during the third trimester as well as one specific organophosphate, chlorpyrifos, during the second trimester. They also report statistically significant associations between ASDs and pyrethroid application both preconception and during the third trimester, as well as an association between carbamate application and developmental delay, although this estimate was based on a small number of cases.^4^

The new research supports associations reported in previous work, says Kirsten Brandt, a senior lecturer at the School of Agriculture, Food, and Rural Development at University of Newcastle, United Kingdom, who was not involved in the study. First author Janie Shelton, a graduate student in the Department of Public Health Sciences at the University of California, Davis, says the most important finding was the association between chlorpyrifos and ASDs. The compound is banned for residential use but is one of the most commonly used agricultural chemicals, she says, noting that pesticides can drift beyond buffer zones around the point of application and into homes and workplaces.^6^

Importantly, however, the study is limited by restrospective data collection and a lack of biological samples collected during gestation. Other limitations include the inability to account for other potential sources of exposure (for instance, pesticides used in the home) as well as multiple exposures, and the possibility of reporting errors in the Pesticide Use Report. It also can be difficult to attribute associations to any specific time window during pregnancy, because exposures may span trimesters—a ubiquitous problem in studying gestational exposures.

Additionally, “It is always a concern when cases and controls have been recruited by different means,” says Philippe Grandjean, an adjunct professor of environmental health at the Harvard School of Public Health, who was not involved in the study. “For example, there are far fewer controls than cases from Southern California.” However, the authors weighted their analysis to at least partially account for differences in the case and control populations.^4^

Despite the limitations, Grandjean adds, “The researchers must be praised for having been able to link data on pesticide usage to geocoded residences during pregnancy.” Joseph Braun, an assistant professor in the Department of Epidemiology at Brown University, describes the work as “the cutting edge of research into environmental determinants of autism.” Braun also was not involved in the study.

Despite the study’s limitations, it provides new directions for exploration. “Until about five years ago, virtually all research on autism assumed that the disease was entirely genetic in origin, and that environmental exposures did not play a role,” says Robert Wright, director of the Division of Environmental Health at Mount Sinai School of Medicine, who was not involved in the research. “Rising rates of autism and failure to find genetic causes despite a multitude of very large genetic studies has led to a major shift in focus in the field. … These chemicals are a solid lead that needs to be followed.”
